# The Korean Frailty and Aging Cohort Study: A Decade of Progress and Future Directions

**DOI:** 10.56786/PHWR.2026.19.41suppl.5

**Published:** 2026-10-29

**Authors:** Jiwon Yi, Yunhwan Lee, Belong Cho, Hak Chul Jang, Yong Soon Park, Ki-Soo Park, Hyeon Ju Kim, Min-Keun Song, Hyuk Jung Kweon, Woo Jung Kim, Eyun Song, Miji Kim, Chang Won Won

**Affiliations:** 1 Elderly Frailty Research Center, Kyung Hee University Seoul Korea; 2 Department of Preventive Medicine and Public Health, Ajou University School of Medicine Suwon Korea; 3 Department of Family Medicine, Seoul National University College of Medicine Seoul Korea; 4 Department of Internal Medicine, Seoul National University Bundang Hospital Seongnam Korea; 5 Department of Family Medicine, Hallym University College of Medicine Chuncheon Korea; 6 Department of Preventive Medicine, College of Medicine, Gyeongsang National University Jinju Korea; 7 Department of Family Medicine, Jeju National University College of Medicine Jeju Korea; 8 Department of Physical and Rehabilitation Medicine, Chonnam National University Medical School, Jeonnam-Gwangju Korea; 9 Department of Family Medicine, Konkuk University Chungju Hospital, Konkuk University College of Medicine Chungju Korea; 10 Department of Psychiatry, Yongin Severance Hospital, Yonsei University College of Medicine Yongin Korea; 11 Division of Endocrinology and Metabolism, Department of Internal Medicine, Korea University College of Medicine Seoul Korea; 12 Department of Health Sciences and Technology, College of Medicine, Kyung Hee University Seoul Korea; 13 Department of Family Medicine, College of Medicine, Kyung Hee University Seoul Korea

**Keywords:** Frailty, Aged, Sarcopenia, Cohort studies

## Abstract

**Objectives**: The Korean Frailty and Aging Cohort Study (KFACS) is a multicenter prospective cohort study investigating frailty, its risk factors, and health outcomes in community-dwelling older adults. It aims to identify prognostic indicators, develop strategies to prevent frailty and disability, and inform future frailty research.

**Methods**: Community-dwelling older adults aged 70–84 years were recruited from 10 university hospitals across the Republic of Korea (ROK) in 2016–2017 and assessed at baseline and biennially thereafter. Standardized assessments collected multidimensional frailty data, including sociodemographic characteristics, health behaviors, chronic diseases, physical and cognitive function, nutritional status, and psychosocial factors.

**Results**: KFACS-based studies have examined frailty prevalence, incidence, progression, and recovery; the relationship between sarcopenia and frailty; and frailty-related health outcomes and prognosis, informing prevention and management strategies.

**Conclusions**: KFACS is a key platform for frailty research in the ROK, with continued advances in frailty prevention and preserving health and function in older adults.

**Key messages**

**① What is known previously?**

Frailty involves age-related declines in physiological reserve and functional capacity, increasing the risk of adverse health outcomes, including functional decline, disability, hospitalization, and mortality. Frailty is dynamic and can worsen or improve over time, underscoring the importance of long-term monitoring and management.

**② What new information is presented?**

Long-term Korean Frailty and Aging Cohort Study (KFACS) data have elucidated frailty transitions and their associations with sarcopenia, physical and cognitive function, disability, and healthcare utilization among older Korean adults. As the KFACS enters its 10th year, it is expanding its research scope by recruiting new participants and collecting digital data, including voice recordings and facial images.

**③ What are implications?**

Integrating data from newly recruited participants with digital data may improve early frailty prediction, advance healthy aging research, and inform frailty prevention and management.

## Introduction

In the Republic of Korea (ROK), as of 2025, the older population aged 65 and over accounts for 20.3% of the total population, and it is projected to increase to exceed 30% in 2036 and 40% in 2050 [1]. Along with this rapid population aging, interest in health in older age is increasing, and maintaining health and function in older adults, as well as preventing functional decline and disability, are becoming important public health tasks. Rather than judging health in older age solely by the presence or absence of disease, the World Health Organization approaches it from the perspective of maintaining functional ability required for daily life, based on an individual’s physical and mental capacities, environment, and the interactions between them [2].

From this perspective, frailty is an important concept for understanding the health and functional status of older adults, as well as for predicting and evaluating future changes. Frailty is distinguished from simple aging resulting from increased age, and can be understood as a vulnerable state wherein reserve capacity across multiple physiological systems declines during the aging process, leading to a reduced ability to respond appropriately to stressors [3]. Therefore, in frailty, even relatively minor changes in health status can lead to a significant deterioration in functional status and can trigger various adverse health outcomes, such as falls, functional decline, disability, hospitalization, and mortality [3].

Frailty is not limited to the physical domain, but has multidimensional characteristics complexly associated with various factors, including nutritional status, cognitive function, mental and psychological factors, and the social environment [4]. Furthermore, because frailty status can transition among robust, pre-frail, and frail over time, longitudinal studies that repeatedly measure health status and related factors in the same participants—rather than merely capturing frailty prevalence at a single point in time—are necessary [5]. Through this approach, the relationships among the occurrence, progression, and recovery processes of frailty, as well as the risk factors affecting them and subsequent health outcomes, can be elucidated. In addition, long-term accumulated data can be utilized as an evidence base for exploring frailty risk factors and prognostic and predictive indicators, for early detection of high-risk populations, and for developing intervention strategies and services to prevent frailty and disability.

Based on these needs, the Korean Frailty and Aging Cohort Study (KFACS) was established to follow community-dwelling older adults longitudinally, characterize frailty status and changes over time, and identify associated risk factors and health outcomes [6]. Through prospective follow-up assessments, the study continuously collects and maintains a range of research resources, including epidemiological and clinical data and human biospecimens. These resources are used to conduct various aging-related studies, including research on prognostic and predictive indicators of frailty and disability, geriatric conditions such as sarcopenia, and associated biomarkers. Ultimately, the study aims to generate scientific evidence to support the prevention of frailty and disability and the development of health policies for older adults by advancing the long-term understanding of changes in health and functional status among older adults in the ROK in response to the country’s transition to a super-aged society.

## Study Overview

### 1. Cohort Study Design and Follow-up Status

KFACS is a multicenter prospective cohort study designed to observe long-term changes in frailty status and associated factors among community-dwelling older adults. In the 2016–2017 baseline survey, community-dwelling older adults aged 70–84 years were recruited through ten research centers across the country, covering urban, suburban, and rural areas. The centers comprised three in Seoul, two in Gyeonggi-do, and one each in Gangwon-do, Chungcheongbuk-do, Jeollanam-do, Gyeongsangnam-do, and Jeju-do (Figure 1). Eligible participants were older adults who had no plans to leave the respective area within the following two years, were able to communicate, and had no history of dementia. Participants were recruited through various channels, including senior welfare centers, public health centers, residential complexes, and outpatient clinics. Quota sampling was used according to age group (70–74, 75–79, and 80–84 years, in a ratio of 6:5:4) and sex (males and females). A total of 3,014 participants took part in the baseline survey: 1,559 in 2016 and 1,455 in 2017 [6]. Subsequently, five participants were excluded because they withdrew consent or had participated more than once, leaving 3,009 participants in the follow-up cohort as of 2026.

Following the baseline survey, follow-up surveys are conducted at 2-year intervals for the same participants. In principle, follow-up surveys are conducted via center visits by participants. However, in cases where center visits are difficult due to mobility or health reasons, home visits are conducted. When both center and home visits are impossible, telephone surveys are conducted. In all survey methods, self-response by the participant is prioritized. If self-response is difficult, the survey method and respondent type are adjusted according to the participant’s health status and participation conditions, such as utilizing proxy interviews (Figure 2). Proxy respondents are limited to spouses, cohabiting blood relatives, or individuals who visit at least once a week to care for the participant (such as neighbors, friends, or caregivers). Through this approach, changes in frailty status and major health outcomes are continuously identified while minimizing loss to follow-up that may occur during the long-term follow-up of older participants.

Starting with the 2016–2017 baseline survey, the first, second, third, and fourth follow-up surveys (Waves 2, 3, 4, and 5, respectively) were sequentially conducted in 2018–2019, 2020–2021, 2022–2023, and 2024–2025, respectively. The follow-up rate was calculated as the proportion of survey completers relative to the target survey population, excluding deaths confirmed in previous surveys. Survey completers did not include participants who did not participate in the survey due to reasons such as refusal to participate in the study or admission to long-term care facilities. The follow-up rates for the first follow-up survey were 92.5% in 2018 and 94.8% in 2019, while the most recently completed fourth follow-up survey recorded rates of 71.5% in 2024 and 74.7% in 2025 (Table 1). Up to 2025, a cumulative total of 500 deaths were confirmed, and the number of follow-up management participants excluding deceased individuals was counted as 2,509.

To ensure consistency and reliability of data collected from multiple centers, interviewers at each research center performed surveys according to standardized survey protocols and manuals after completing training provided by the central center (Laboratory of Frailty and Aging Epidemiology, Kyung Hee University Hospital). Furthermore, continuous quality control and monitoring were conducted throughout the entire survey process, and participants’ follow-up status—such as death, health deterioration, relocation, and discontinuation of participation—was systematically managed by survey wave [6].

### 2. Cohort Survey Items and Establishment of Research Resources

To comprehensively identify factors affecting the occurrence and status changes of frailty, various types of information encompassing physical, cognitive, and psychosocial domains were repeatedly collected (Figure 3). The main survey items consisted of frailty and physical function, health status and healthcare utilization, health behaviors and nutrition, social functioning, cognitive function, and sociodemographic characteristics. Along with questionnaire surveys, physical measurements, physical performance tests, clinical examinations, and blood/urine tests were conducted to comprehensively evaluate participants’ health and functional status (Tables 2, 3) [6].

Detailed items of the questionnaire and health examination surveys were applied differentially according to the survey method and respondent type (Table 4). In the case of participant self-response during center visits, the entire questionnaire survey and health examination survey were conducted, whereas in the case of proxy response, only certain questionnaire items were surveyed. During home visits, participant self-response included the entire questionnaire survey and certain examination items, whereas proxy response covered only the same questionnaire items as proxy responses during center visits. In telephone surveys, both self-response and proxy response surveyed only certain questionnaire items. Across all three survey methods, health examination surveys were not conducted in the case of proxy responses.

Frailty, a primary outcome measure, was assessed using Fried’s frailty phenotype from the Cardiovascular Health Study, which evaluates five components: unintentional weight loss, exhaustion, low physical activity, slow walking speed, and weakness. Under the modified diagnostic criteria, participants are classified as frail if they meet three or more components, pre-frail if they meet one or two components, and robust if they meet none [6]. In addition, various frailty assessment tools were used in this study, including the FRAIL questionnaire and the Korean Frailty Index, which was developed for use in this study. Sarcopenia screening tools, including SARC-F, are also employed. Repeated collection of data on physical function, cognitive function, clinical information, and human biospecimens enables longitudinal analyses of individual health changes and factors associated with frailty. More recently, the scope of research resources has been expanded to include domains such as digital device literacy and oral frailty, as well as digitally acquired measurements such as voice and facial expressions [7].

### 3. Major Research Achievements of the KFACS Cohort

Studies using baseline and follow-up data from KFACS have investigated various domains related to frailty among community-dwelling older adults in the ROK, including frailty prevalence and changes in frailty status, sarcopenia, physical and cognitive function, and healthcare utilization.

#### 1) Frailty prevalence and frailty status transitions

Standardizing the data to reflect the ROK’s population structure among 2,907 baseline survey participants in 2016–2017 who completed the Fried physical frailty phenotype assessment, the prevalence of frailty was 7.9% (95% confidence interval [CI], 6.8–8.9%) and the prevalence of pre-frailty was 47.0% (95% CI, 45.1–48.8%) [8]. Frailty prevalence showed a trend of increasing with age, and various factors were associated with frailty, including nutritional risk, sarcopenia, mobility limitation, depressive symptoms, polypharmacy, low social capital, and low physical performance [8].

In particular, frailty exhibits dynamic characteristics that can change over time among robust, pre-frail, and frail states. Systematic literature reviews and meta-analyses have also reported transitions improving to previous stages as well as cases where frailty status worsens [5]. In analyses utilizing long-term follow-up data from KFACS, maintenance, worsening, and improvement of frailty status were all observed [9], and recently, changes in intrinsic capacity were shown to be associated with the persistence or worsening of frailty status. Specifically, declines in mobility, vitality, and psychological domains were related to worsening frailty status, suggesting the necessity to consider various functional domains together [10].

An internal analysis of the 8-year follow-up data accumulated up to 2024 also confirmed long-term status transitions. Comparing the 2016 baseline survey with the 2024 follow-up survey, the incidence of new frailty was 11.8%, and the frailty prevalence increased from 8.8% to 14.1%. Regarding frailty status transitions over 8 years, 50% maintained their original status, whereas 33% worsened and 17% improved; in pre-frailty, the proportion recovering to a robust state was relatively higher than transitioning to a frail state [7]. These results demonstrate that it is important to identify risk factors early and implement appropriate preventive management before frailty worsens, showing the potential to prevent or delay the progression of frailty.

#### 2) Research related to sarcopenia

Sarcopenia is a representative geriatric syndrome associated with declines in muscle mass, muscle strength, and physical performance, and is closely related to frailty. In a study analyzing 2,028 KFACS participants, the prevalence of sarcopenia based on the Asian Working Group for Sarcopenia 2019 criteria was 17.5%, and participants with co-occurring frailty exhibited fall-related health problems, such as recurrent falls and fall-related fractures, more frequently than those with only sarcopenia or only frailty [11]. This finding indicates that while frailty and sarcopenia share some characteristics, they also possess distinct clinical features.

KFACS has also been used to examine the application of international diagnostic criteria for sarcopenia and to evaluate screening and assessment methods for community-dwelling older adults in the ROK. A study applying the European Working Group on Sarcopenia in Older People criteria found that the prevalence of sarcopenia varied according to the assessment methods and clinical algorithms used [12]. A study using calf circumference proposed cutoff values of 35 cm for males and 33 cm for females to screen for low muscle mass [13]. In addition, the validity of the Korean version of SARC-F was established [14], and the five-times sit-to-stand test was reported to be a convenient supplementary measure for assessing muscle strength and physical performance in the diagnosis of sarcopenia [15].

The research scope has expanded to include sarcopenic obesity, its relationship with physical function, screening methods for sarcopenic obesity [16,17], and the relationship between blood-based biomarkers and muscle function [18]. Recently, studies for early screening and prediction of sarcopenia are also being conducted, such as developing possible sarcopenia prediction models using machine learning techniques on accumulated clinical and functional data [19]. These studies demonstrate that KFACS is being utilized as a research foundation capable of conducting screening, risk factor, and prediction research as well as epidemiological studies on sarcopenia.

#### 3) Major health outcomes and prognostic research related to frailty

Studies using KFACS follow-up data have examined the associations between frailty and major subsequent health outcomes, including functional decline and disability. Analyses of baseline and two-year follow-up data found that physical frailty was associated with the subsequent development of mobility disability and disability in instrumental activities of daily living. The risk of mobility disability was also elevated among participants classified as pre-frail [20]. Furthermore, participants who performed poorly on the five-times sit-to-stand test had a higher risk of adverse health outcomes at follow-up, including slower gait speed and functional limitations, suggesting the potential prognostic value of physical performance measures [21]. The Timed Up & Go test was also found to have potential as a simple screening tool for physical frailty and low physical performance [22].

KFACS is also being utilized in research across other health domains, including cognitive function and healthcare utilization. A study analyzing the relationship between gait speed and cognitive function showed that slow gait speed was associated with low cognitive function, particularly frontal lobe–related function [23], and a 2-year longitudinal study demonstrated that high psychological resilience was associated with less decline in overall cognitive function [24]. In addition, a study linking KFACS with National Health Insurance data showed that the group whose condition worsened from pre-frail to frail over 2 years had increased hospital stay days and medical costs compared to the group that maintained a robust state [25]. These findings demonstrate that KFACS is utilized as a foundation to study changes in health status and prognosis in older age across various domains, including physical function and disability, as well as cognitive function and healthcare utilization.

### 4. Future of the Cohort Study

While continuing the long-term follow-up of existing participants, KFACS is recruiting approximately 800 new community-dwelling older adults aged 70–84 years in 2026–2027. This recruitment aims to address the declining number of participants available for follow-up due to cohort aging and associated factors, including deaths, deteriorating health, and admission to long-term care facilities, while also investigating the characteristics of changing older generations [7]. By applying an age-group distribution and assessment framework similar to those of the existing cohort, the study aims to establish a basis for comparing frailty and health characteristics between two groups entering older age approximately 10 years apart and for analyzing generational effects associated with changes in social and environmental factors and lifestyle.

Moving beyond existing epidemiological and clinical data and human biospecimen-centered research, the research domain is expanding to incorporate digital biomarkers such as voice and facial video data. Recent studies have reported that the acoustic characteristics of voice may be associated with frailty, and research is also underway to classify frailty by applying artificial intelligence to voice data [26,27]. In addition, the value of facial video as a non-invasive digital indicator is gaining attention, as evidenced by studies analyzing the association between facial expression features extracted from facial video and cognitive impairment, as well as research attempting early screening of dementia using artificial intelligence–based facial expression analysis [28,29]. In KFACS as well, we aim to explore the potential utilization of features extracted from voice and facial video as new digital indicators that can assess and predict frailty and functional decline based on existing physical/cognitive function and clinical data.

This expansion of research may contribute to the analysis of long-term changes in the existing cohort and the characteristics of newly recruited generations, as well as to the identification of factors and predictive indicators influencing the onset and progression of frailty. Furthermore, linking clinical and epidemiological data, human biospecimens, and digital data is expected to build an evidence base for the early identification of individuals at high risk of frailty and the development of prevention and management strategies. These efforts may also help elucidate intergenerational changes in frailty and health characteristics and expand the scope of aging research through new approaches that integrate digital data with existing clinical and epidemiological data and human biospecimens.

## Conclusion

KFACS has established a research foundation that can identify the incidence and changes of frailty, related risk factors, and health outcomes among community-dwelling older adults in the ROK through long-term repeated follow-ups. Epidemiological and clinical data, along with human biospecimens accumulated through continuous follow-up surveys, are widely used to study not only frailty, but also sarcopenia, physical and cognitive functions, and various health problems in older age. Going forward, by continuing long-term follow-up of existing participants, recruiting new subjects, and utilizing newly constructed research resources, KFACS is expected to continue its role as a research foundation that enables long-term observation and understanding of the changing health and frailty patterns of older adults in the ROK.

## Figures and Tables

**Figure 1:**
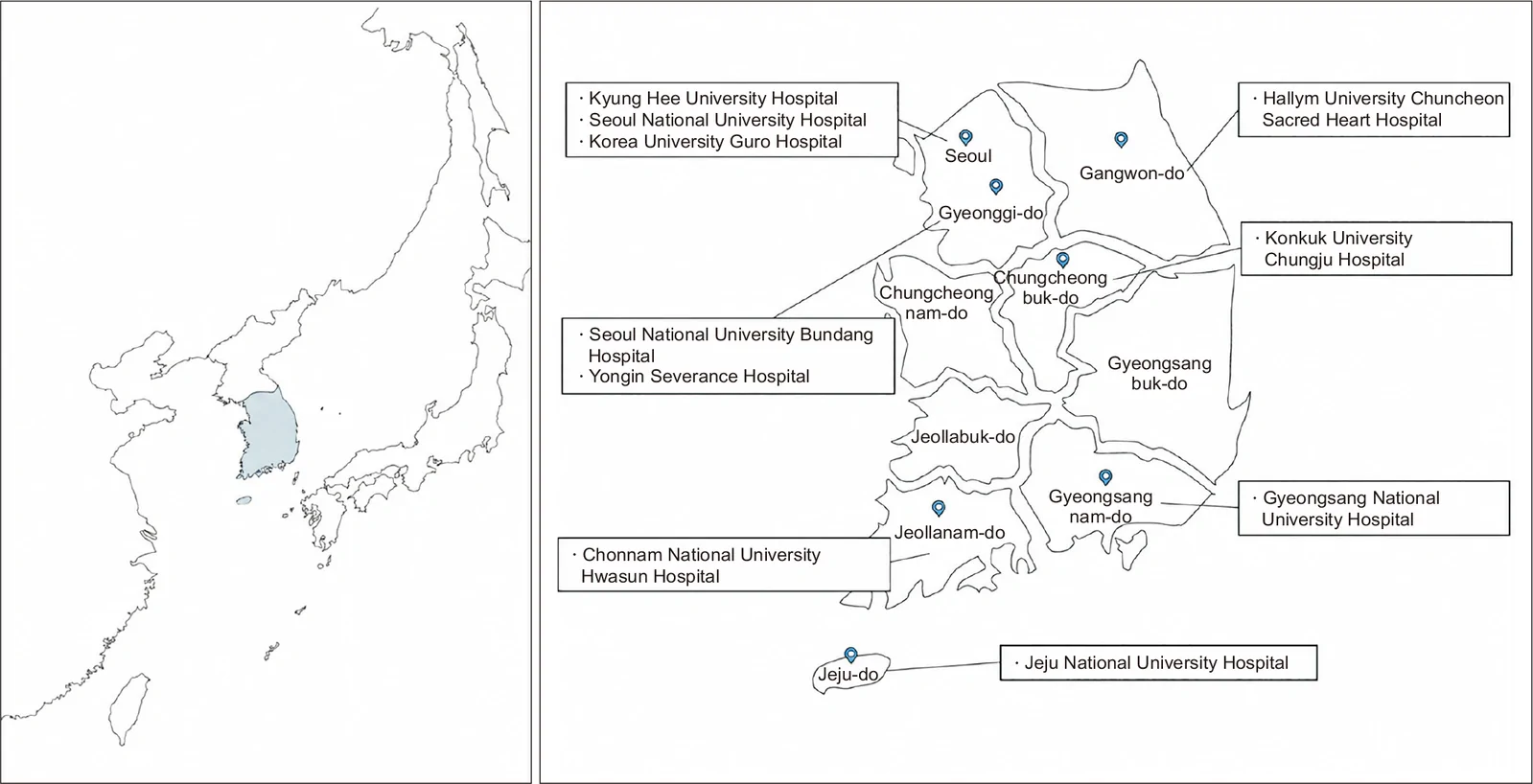
Locations of the 10 centers involved in the Korean Frailty and Aging Cohort Study

**Figure 2:**
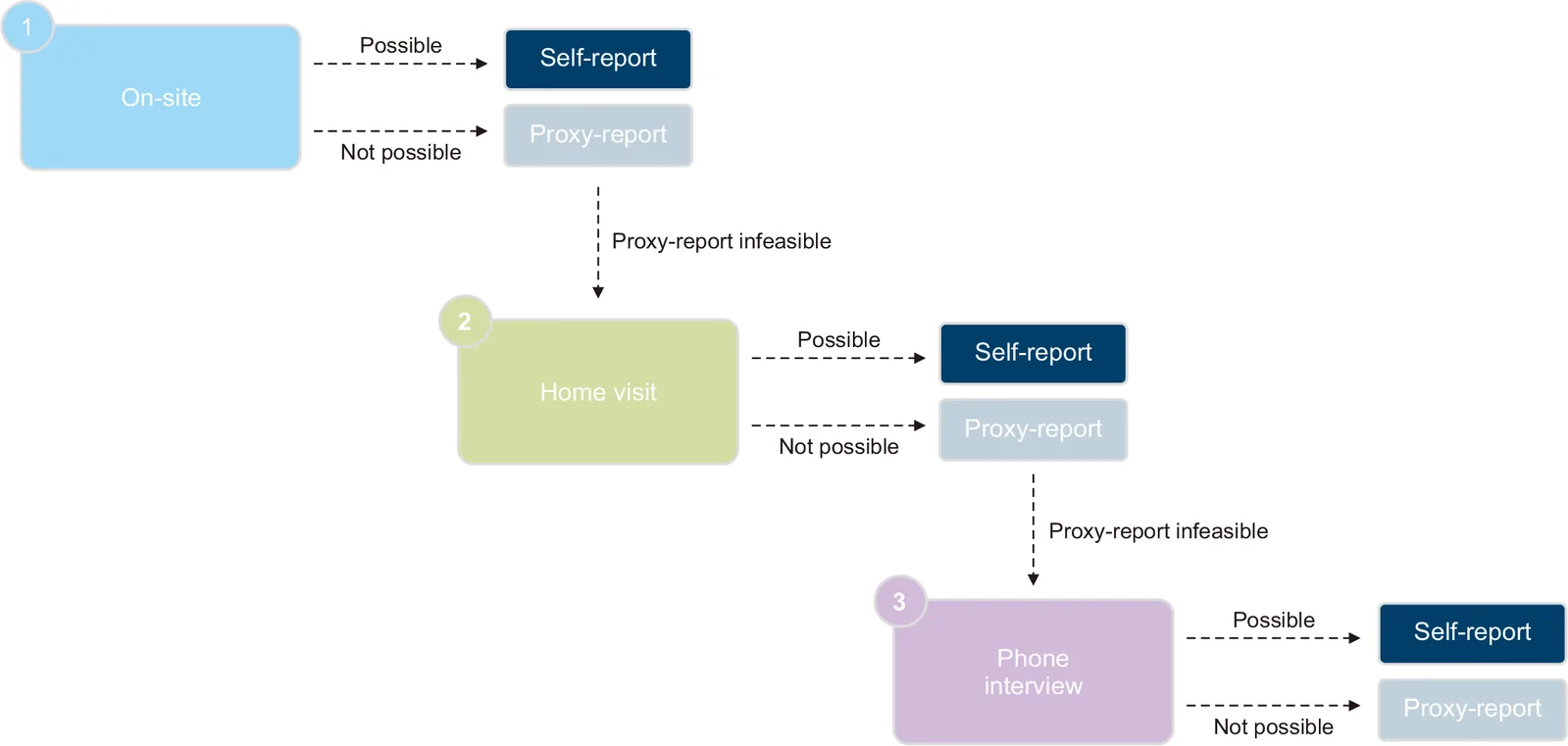
Methods for follow-up assessments in the Korean Frailty and Aging Cohort Study

**Figure 3:**
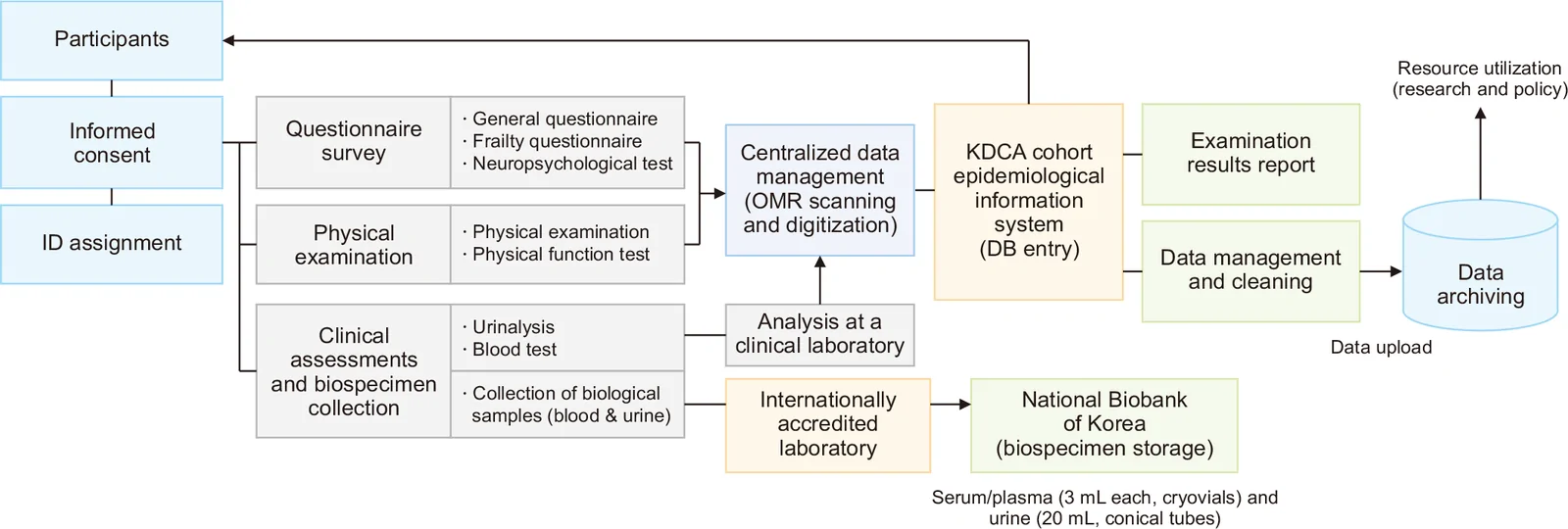
Epidemiological data and biospecimen collection and management in the KFACS follow-up study ID=identification; OMR=optical mark recognition; KDCA=Korea Disease Control and Prevention Agency; DB=database; KFACS= Korean Frailty and Aging Cohort Study.

**Table 1:** Follow-up status and rates in the Korean Frailty and Aging Cohort Study

Study wave	Year	Subjects (n)	Follow-up (n)	Follow-up rate (%)	Deaths (n)
BL	2016	1,558a)	-	-	-
	2017	1,453b)	-	-	-
Wave 2	2018	1,558	1,441	92.5	18
	2019	1,453	1,377	94.8	20
Wave 3	2020	1,540	1,347	87.5	45
	2021	1,433	1,112	77.6	41
Wave 4	2022	1,495	1,135	75.9	105
	2023	1,392	1,106	79.5	63
Wave 5	2024	1,390	994	71.5	115
	2025	1,327c)	991	74.7	93

BL=baseline. ^a)^Of the 1,559 participants enrolled at baseline in 2016, one withdrew consent, leaving 1,558 participants. ^b)^Of the 1,455 participants enrolled at baseline in 2017, two withdrew consent, leaving 1,453 participants. ^c)^In 2025, two additional participants from the 2017 baseline cohort withdrew consent, resulting in 1,327 eligible participants after excluding previous deaths.

**Table 2:** Korean Frailty and Aging Cohort Study questionnaire items

Category	Variable
1. Basic information	Participant information, assessment method (center visit, home visit, telephone interview), NHIS data, subjective age
2. Frailty assessment	CHS (Fried phenotype), KLoSHA Frailty Index (K-IADL, K-ADL), FRAIL, KFI, Frailty Scale, SOF Frailty Index, physical functional limitations (PF scale-mobility), SARC-F, KFS
3. Health status	Morbidity, prior and concomitant medications, long-term care insurance and healthcare utilization, community health and welfare service utilization, health literacy, quality of life (EQ-5D/EQ-VAS/SF-12), mental health (SGDS-K/BRS/LOT-R/UCLA Loneliness Scale/PSS/PSQI/The [Secure] Flourish Index), experience of falls, LF scale
4. Health behaviour	Smoking, alcohol drinking, IPAQ, nutrition (MNA/SNAQ/appetite/eating behavior), oral health, women’s health
5. Social function	Social network, social capital, social support, frequency of going out
6. Cognitive function	MMSE-KC, memory (word list memory/word list recall/word list recognition), TMT-A, attention (digit span forward/backward), FAB
6-1. Cognitive function (※only for proxy interviews)	K-AD8 (proxy)
7. Demographics	Education, work/employment, marital status, household characteristics, National Basic Livelihood Security beneficiary status, mobile phone ownership, subjective economic status, MDPQ-16

NHIS=National Health Insurance Service; CHS=Cardiovascular Health Study; KLoSHA=Korean Longitudinal Study on Health and Aging; K-IADL=Korean Instrumental Activities of Daily Living; K-ADL=Korean Activities of Daily Living; FRAIL=Fatigue, Resistance, Ambulation, Illness, Loss of Weight; KFI=Korean Frailty Index; SOF=Study of Osteoporotic Fractures; PF=Physical Function; SARC-F=Strength, Assistance in walking, Rise from a chair, Climb stairs, and Falls; KFS=Korean Frailty Scale; EQ-5D=EuroQol 5-Dimension; EQ-VAS=EuroQol Visual Analogue Scale; SF-12=12-Item Short Form Health Survey; SGDS-K=Korean version of the Short Form of Geriatric Depression Scale; BRS=Brief Resilience Scale; LOT-R=Life Orientation Test-Revised; UCLA Loneliness Scale=University of California, Los Angeles Loneliness Scale; PSS=Perceived Stress Scale; PSQI=Pittsburgh Sleep Quality Index; LF=Life Functioning; IPAQ=International Physical Activity Questionnaire; MNA=Mini Nutritional Assessment; SNAQ=Simplified Nutritional Appetite Questionnaire; MMSE-KC=Mini-Mental Status Examination, Korean version of CERAD Assessment Packet; TMT-A=Trail Making Test A; K-AD8=Korean version of the Ascertain Dementia 8-item Informant Questionnaire; FAB=Frontal Assessment Battery; MDPQ-16=Mobile Device Proficiency Questionnaire.

**Table 3:** Korean Frailty and Aging Cohort Study examination items

Category	Variable
Physical examination	
1. Anthropometry	Body weight/height/waist circumference/upper arm circumference/calf circumference/finger-ring test
2. Vital signs	Blood pressure (SBP/DBP), mmHg/heart rate
3. Visual acuity and hearing	Visual acuity - Distance visual acuity/near visual acuity/stereoacuity test (Titmus Fly Test) Hearing - 500/1,000/2,000/4,000 Hz
4. Blood collection	Glucose (FBS), insulin, HbA1c, total protein, albumin, BUN, creatinine, GOT (AST), GPT (ALT), GGT (γ-GTP), total cholesterol, HDL-cholesterol, LDL-cholesterol, triglyceride, uric acid, WBC, RBC, Hb, Hct, platelet, MCV, MCH, MCHC, (analyses added in 2024: hs-CRP, cystatin C)
5. Urine collection	pH, nitrite, S.G, protein, glucose, ketone, bilirubin, occult blood, urobilinogen, leukocyte esterase
6. ECG	
7. Chest X-ray	
8. Oral frailty	Oral frailty risk screening questionnaire (12 items)
9. Body composition	DXA/BIA
10. Bone mineral density	DXA (femur and lumbar spine) (examinations added in 2024)
Physical function	
1. Hand grip strength	
2. Short Physical Performance Battery (SPPB)	Standing balance (side-by-side stance/semi-tandem stance/tandem stance) Usual gait speed - 4 m usual gait speed (with acceleration/deceleration zones) (sensor/stopwatch measurement) - 4 m usual gait speed (without acceleration/deceleration zones) (sensor/stopwatch measurement) - 2.4 m usual gait speed (without acceleration/deceleration zones) (stopwatch measurement [home visit]) Chair-stand time - Stopwatch measurement
3. Timed Up & Go test (3 m walk and return)	
4. Leg muscle strength	Knee extension strength (2 trials)/knee flexion strength (2 trials)
5. Gait measurement	
6. Voice	ODK performance/standard passage reading/spontaneous speech (picture description)
7. Facial	Resting facial video/speech-facial video

SBP=systolic blood pressure; DBP=diastolic blood pressure; FBS=fasting blood sugar; HbA1c=hemoglobin A1c; BUN=blood urea nitrogen; GOT=glutamic oxaloacetic transaminase; AST=aspartate aminotransferase; GPT=glutamic pyruvic transaminase; ALT=alanine aminotransferase; GGT=gamma-glutamyl transferase; γ-GTP=gamma-glutamyl transpeptidase; HDL=high-density lipoprotein; LDL=low-density lipoprotein; WBC=white blood cell; RBC=red blood cell; Hb=hemoglobin; Hct=hematocrit; MCV=mean corpuscular volume; MCH=mean corpuscular hemoglobin; MCHC=mean corpuscular hemoglobin concentration; hs-CRP=high-sensitivity C-reactive protein; S.G=specific gravity; ECG=electrocardiography; DXA=dual-energy X-ray absorptiometry; BIA=bioelectrical impedance analysis; ODK=oral diadochokinesis.

**Table 4:** Summary of assessment items by the follow-up method

Survey method	Assessment type	Self-report	Proxy
Center visit	Questionnaire	Full questionnaire items	Selected questionnaire items (cohabitation status, relationship to participant, participant’s current status, Korean Instrumental Activities of Daily Living [K-IADL], FRAIL, falls, health behavior [smoking, alcohol drinking, physical activity, and nutrition], social function [social capital], cognitive function [K-AD8], demographics [marital status, household composition, household income])
	Examination	Full examination items	-
Home visit	Questionnaire	Full questionnaire items	Same as center-visit proxy respondents
	Examination	Selected examination items (anthropometry [body weight/height, body circumference], vital signs [blood pressure/heart rate], visual acuity and hearing, blood collection, physical function [hand grip strength, SPPB])	-
Phone interview	Questionnaire	Selected questionnaire items (K-IADL, K-ADL, FRAIL, experience of falls, body weight/height, body weight last year)	Selected questionnaire items (cohabitation status, relationship to participant, participant’s current status, K-IADL, FRAIL, experience of falls, K-AD8)
	Examination	-	-

FRAIL=Fatigue, Resistance, Ambulation, Illness, Loss of Weight; K-AD8=Korean version of the Ascertain Dementia 8-item Informant Questionnaire; SPPB=Short Physical Performance Battery; K-ADL=Korean Activities of Daily Living.
