# Supplementary material for: The Korean Frailty and Aging Cohort Study: A Decade of Progress and Future Directions
Source: Public Health Wkly Rep. 2026 Oct 29;19(41 Suppl):83–106. doi: 10.56786/PHWR.2026.19.41suppl.5 (PMC13725165; doi:10.56786/PHWR.2026.19.41suppl.5)
Supplement: Translation

# Translation: 한국 노인노쇠코호트 연구: 10년간의 성과와 향후 연구 방향

**Authors:** 지원 이, 윤환 이, 비룡 조, 학철 장, 용순 박, 기수 박, 현주 김, 민근 송, 혁중 권, 우정 김, 의연 송, 미지 김, 장원 원

**Affiliations:** 1 경희대학교 노인노쇠-근감소 연구센터; 2 아주대학교 의과대학 예방의학교실; 3 서울대학교 의과대학 가정의학교실; 4 분당서울대학교병원 내과; 5 한림대학교 의과대학 가정의학교실; 6 경상국립대학교 의과대학 예방의학교실; 7 제주대학교 의과대학 가정의학교실; 8 전남대학교 의과대학 재활의학교실; 9 건국대학교 의과대학 건국대학교충주병원 가정의학과; 10 연세대학교 의과대학 용인세브란스병원 정신건강의학과; 11 고려대학교 의과대학 내과학교실 내분비내과; 12 경희대학교 의과대학 의예과; 13 경희대학교 의과대학 가정의학교실

**Keywords:** 노쇠, 노인, 근감소증, 코호트 연구

## Abstract

**목적:**

한국 노인노쇠코호트 연구(Korean Frailty and Aging Cohort Study, KFACS)는 지역사회 거주 노인의 노쇠 상태와 변화 양상을 장기적으로 추적하고, 노쇠의 위험요인과 건강결과를 규명하기 위해 구축된 다기관 전향적 코호트 연구이다. 본 연구는 노쇠 및 장애 예방을 위한 예후‧예측 지표와 중재 방안을 개발하고 향후 노쇠 연구의 기반을 마련하는 것을 목표로 한다.

**방법:**

2016–2017년 전국 10개 대학병원을 기반으로 70–84세 지역사회 거주 노인을 모집하여 기반조사를 수행하고, 이후 2년마다 추적조사를 시행하여 노쇠 상태와 건강 변화를 지속적으로 관찰하고 있다. 표준화된 방법을 통해 인구사회학적 특성, 건강행태, 만성질환, 신체‧인지기능, 영양 상태 및 심리‧사회적 요인 등 다차원적 자료를 수집하고 있다.

**결과:**

노쇠의 유병률과 발생률을 제시하고, 노쇠의 진행 및 회복과 관련된 주요 요인을 규명해 왔다. 또한 근감소증과 노쇠의 연관성, 노쇠에 따른 주요 건강결과와 예후를 분석함으로써 노쇠의 예방과 관리에 필요한 근거를 축적하였다.

**결론:**

KFACS는 국내 노쇠 연구의 주요 연구 기반으로서, 지속적인 추적조사와 자료의 활용을 통해 노쇠 예방 및 노인의 건강과 기능 유지를 위한 근거 마련에 기여할 것으로 기대된다.

**핵심요약**

**① 이전에 알려진 내용은?**

노쇠는 노화에 따른 생리적 예비능력과 기능의 저하로 발생하며, 기능 저하, 장애, 입원 및 사망 등 다양한 부정적 건강결과의 위험을 높이는 것으로 알려져 있다. 또한 노쇠는 고정된 상태가 아니라 시간에 따라 악화되거나 회복될 수 있어 장기적인 추적과 관리가 중요하다.

**② 새로이 알게 된 내용은?**

한국 노인노쇠코호트 연구(Korean Frailty and Aging Cohort Study, KFACS) 장기 추적을 통해 국내 노인의 노쇠 이행과 근감소증, 신체‧인지기능, 장애 및 의료이용 등 다양한 건강결과와의 관련성을 확인하였으며, 최근에는 KFACS의 10년을 맞이하여 신규 대상자 모집과 음성‧얼굴 영상 등 디지털 자료 수집을 통해 연구 범위를 확대하고 있다.

**③ 시사점은?**

향후 신규 코호트와 디지털 자료를 연계하여 노쇠의 조기 예측과 건강노화 연구를 확대하고, 노쇠 예방 및 관리에 활용할 수 있을 것으로 기대된다.

## 서 론

우리나라는 2025년 기준 65세 이상 고령인구가 전체 인구의 20.3%를 차지하며, 향후에도 지속적으로 증가하여 2036년에는 30%, 2050년에는 40%를 넘어설 것으로 전망된다[1]. 이처럼 급속한 인구 고령화와 함께 노년기 건강에 대한 관심이 증가하고 있으며, 노인의 건강과 기능을 유지하고 기능 저하와 장애를 예방하는 것은 중요한 보건학적 과제가 되고 있다. 세계보건기구(World Health Organization)는 노년기 건강한 노화를 질병의 유무만으로 판단하기보다 개인의 신체적‧정신적 능력과 환경 및 이들 간의 상호작용을 바탕으로 일상생활에 필요한 기능적 능력(functional ability)을 유지하는 관점에서 접근하고 있다[2].

이러한 관점에서 노쇠(frailty)는 고령자의 건강과 기능 상태를 이해하고 향후 변화를 예측‧평가하는 데 중요한 개념이다. 노쇠는 단순한 연령 증가에 따른 노화와는 구별되며, 노화 과정에서 여러 생리적 체계의 예비능력이 감소하면서 스트레스 요인에 적절히 대응하는 능력이 저하된 취약한 상태로 이해할 수 있다[3]. 따라서, 노쇠는 비교적 작은 건강상의 변화에도 기능 상태가 크게 악화될 수 있으며, 낙상, 기능 저하, 장애, 입원 및 사망 등 다양한 부정적 건강결과를 유발할 수 있다[3].

노쇠는 신체적 영역에만 국한되지 않고 영양 상태, 인지기능, 정신‧심리적 요인 및 사회적 환경 등 다양한 요인이 복합적으로 관련되는 다차원적 특성을 가진다[4]. 또한 노쇠는 시간에 따라 건강(robust)-전노쇠(pre-frail)-노쇠(frail) 사이에서 상태가 변화할 수 있으므로 단일 시점의 노쇠 유병 상태를 파악하는 데 그치지 않고, 동일한 대상자의 건강 상태와 관련 요인을 반복적으로 측정하는 종단연구가 필요하다[5]. 이를 통해 노쇠의 발생‧진행‧회복 과정과 이에 영향을 미치는 위험요인 및 이후 건강결과 간의 관계를 규명할 수 있다. 또한 장기간 축적된 자료는 노쇠 위험요인과 예후‧예측 지표를 탐색하고, 고위험군의 조기 발견과 노쇠 및 장애 예방을 위한 중재 전략과 서비스 개발의 근거로 활용될 수 있다.

이러한 필요성을 바탕으로 한국 노인노쇠코호트 연구(Korean Frailty and Aging Cohort Study, KFACS)는 지역사회 거주 노인을 장기간 추적하여 노쇠 상태와 변화 양상을 파악하고 관련 위험요인 및 건강결과를 규명하기 위해 구축되었다[6]. 전향적 추적조사를 통해 역학‧임상정보와 인체자원 등 다양한 연구자원을 지속적으로 확보하고 있으며, 이를 활용하여 노쇠 및 장애의 예후‧예측 지표뿐 아니라, 근감소증(sarcopenia) 등의 노인성 질환과 관련 바이오마커 등 다양한 노화 연구를 수행하고 있다. 궁극적으로는 초고령사회에 대응하여 국내 노인의 건강과 기능 변화를 장기적으로 이해하고, 노쇠 및 장애 예방과 노인 건강정책 수립에 필요한 과학적 근거를 마련하는 것을 목표로 한다.

## 본 론

### 1. 코호트 연구 설계 및 추적 현황

KFACS는 지역사회 거주 노인의 노쇠 상태와 관련 요인의 변화를 장기간 관찰하기 위한 다기관 전향적 코호트 연구이다. 2016–2017년 기반조사(baseline)에서는 전국의 도시, 교외 및 농촌지역을 포함한 10개 연구센터를 중심으로 70–84세 지역사회 거주 노인을 모집하였다. 연구센터는 서울 3개소, 경기도 2개소, 강원도‧충청북도‧전라남도‧경상남도‧제주도 각 1개소로 구성되었다(그림 1). 연구 대상자는 향후 2년 이내 해당 지역을 떠날 계획이 없고 의사소통이 가능하며, 치매 진단 과거력이 없는 노인으로 선정하였다. 노인복지관, 보건소, 주거단지 및 외래진료기관 등 다양한 경로를 통해 대상자를 모집하였으며, 연령(70–74세, 75–79세, 80–84세; 6:5:4의 비율)과 성별(남성 및 여성)에 따른 할당표본추출(quota sampling)을 적용하였다. 그 결과, 2016년 1,559명, 2017년 1,455명 등 총 3,014명이 기반조사에 참여하였으며[6], 이후 동의 철회 및 중복 참여 등으로 5명이 제외되어 2026년 기준으로 최종 추적관리 대상자는 총 3,009명이다.

기반조사 이후 동일한 대상자를 대상으로 2년 간격의 추적조사를 시행하고 있다. 추적조사는 대상자의 센터방문을 원칙으로 하며, 거동이나 건강상의 이유로 센터방문이 어려운 경우에는 가구방문을 실시하고, 센터 및 가구방문이 모두 불가능한 경우에는 전화조사를 실시한다. 모든 조사 방법에서 대상자 본인의 응답을 우선으로 하며, 본인응답이 어려운 경우에는 대리응답자 조사(proxy interview)를 활용하는 등 대상자의 건강 상태와 조사 참여 여건에 따라 조사 방법 및 응답자 유형을 조정하고 있다(그림 2). 대리응답자는 배우자, 동거 중인 혈연관계인 또는 일주일에 1회 이상 대상자를 돌보기 위해 방문하는 사람(이웃, 친구, 간병인 등)으로 제한한다. 이를 통해 고령 대상자의 장기 추적 과정에서 발생할 수 있는 추적 손실을 최소화하면서 노쇠 상태와 주요 건강결과의 변화를 지속적으로 파악하고 있다.

2016–2017년 기반조사를 시작으로 2018–2019년 1차 추적조사(Wave 2), 2020–2021년 2차 추적조사(Wave 3), 2022–2023년 3차 추적조사(Wave 4), 2024–2025년 4차 추적조사(Wave 5)가 순차적으로 수행되었다. 추적률은 이전 조사에서 확인된 사망자를 제외한 조사 대상자 대비 조사 완료자의 비율로 산정하였으며, 조사 완료자에는 연구 참여 거부, 요양시설 입소 등의 사유로 조사에 참여하지 않은 대상자를 포함하지 않았다. 1차 추적조사의 추적률은 2018년 92.5%, 2019년 94.8%였으며, 가장 최근 완료된 4차 추적조사에서는 2024년 71.5%, 2025년 74.7%를 기록하였다(표 1). 2025년까지 총 500명의 누적 사망자가 확인되었으며, 사망자를 제외한 추적관리 대상자는 2,509명으로 집계되었다.

다기관에서 수집되는 자료의 일관성과 신뢰도를 확보하기 위해 각 연구센터의 조사자는 중앙센터(경희대학교병원 노화역학연구실)에서 실시하는 교육을 이수한 후 표준화된 조사 프로토콜과 매뉴얼에 따라 조사를 수행한다. 또한 조사 과정 전반에 걸쳐 지속적인 질 관리와 모니터링을 실시하고 있으며, 사망, 건강 악화, 거주지 이전 및 참여 중단 등 대상자의 추적 상태를 조사 차수별로 체계적으로 관리하고 있다[6].

### 2. 코호트 조사 항목 및 연구자원 구축

노쇠의 발생과 상태 변화에 영향을 미치는 요인을 다각적으로 파악하기 위해 신체적‧인지적‧심리사회적 영역을 포괄하는 다양한 정보를 반복적으로 수집하고 있다(그림 3). 주요 조사 항목은 노쇠 및 신체기능, 건강 상태와 의료이용, 건강행태와 영양, 사회적 기능, 인지기능 및 인구‧사회‧경제학적 특성 등으로 구성되며, 설문조사와 함께 신체계측, 신체기능검사, 임상검사 및 혈액‧소변검사 등을 시행하여 대상자의 건강과 기능 상태를 종합적으로 평가하고 있다(표 2, 3) [6].

설문조사 및 검진조사의 세부 항목은 조사 방법과 응답자 유형에 따라 차등 적용된다(표 4). 센터방문 시 대상자 본인응답의 경우 전체 설문조사와 검진조사를 실시하며, 대리응답의 경우 일부 설문 항목만 조사한다. 가구방문 시에는 본인응답의 경우 전체 설문조사와 일부 검진 항목을 실시하며, 대리응답의 경우 센터방문 대리응답과 동일한 설문 항목만 조사한다. 전화조사의 경우 본인응답과 대리응답 모두 일부 설문 항목만 조사한다. 세 가지 조사 방법 모두 대리응답의 경우 검진조사는 실시하지 않는다.

주요 평가 항목인 노쇠는 Fried의 Cardiovascular Health Study 노쇠 표현형을 기반으로 비의도적 체중 감소(weight loss), 탈진(exhaustion), 신체활동 감소(low activity), 보행속도 감소(slowness), 근력 약화(weakness)의 다섯 가지 요소를 평가하여 이 중에서 3개 이상에 해당하는 경우를 노쇠, 1–2개에 해당하는 경우를 전노쇠, 그리고 하나도 해당하지 않을 경우 건강으로 분류하는 수정된 진단 기준을 적용하고 있다[6]. 또한 FRAIL 설문과 함께 Korean Frailty Index를 개발해 사용하는 등 다양한 노쇠 평가도구와 SARC-F를 포함한 근감소증 관련 선별도구도 함께 활용하고 있다. 신체기능과 인지기능, 임상정보 및 인체자원을 반복적으로 축적함으로써 개인별 건강 변화와 노쇠 관련 요인을 종단적으로 분석할 수 있다. 최근에는 디지털 기기 이해력, 구강노쇠 등의 영역과 음성 및 얼굴표정 등 디지털 기반 측정자료를 추가하여 연구자원의 범위를 확대하고 있다[7].

### 3. KFACS의 주요 연구성과

KFACS의 기반조사 및 추적자료를 활용하여 국내 지역사회 거주 노인의 노쇠 유병과 상태 변화, 근감소증, 신체‧인지기능 및 의료이용 등 노쇠와 관련된 다양한 영역에서 연구가 이루어져 왔다.

#### 1) 노쇠 유병률 및 노쇠 상태의 이행

2016–2017년 기반조사 참여자 중 Fried 신체노쇠 표현형 평가가 완료된 2,907명을 대상으로 우리나라 인구구조를 반영하여 표준화한 결과, 노쇠 유병률은 7.9% (95% confidence interval [CI], 6.8–8.9%), 전노쇠 유병률은 47.0% (95% CI, 45.1–48.8%)로 나타났다[8]. 노쇠 유병률은 연령이 증가할수록 높아지는 경향을 보이며, 영양위험, 근감소증, 이동능력 제한, 우울증상, 다약제 복용, 낮은 사회적 자본 및 낮은 신체수행능력 등 다양한 요인이 노쇠와 관련되어 있었다[8].

특히 노쇠는 건강, 전노쇠, 노쇠 상태 사이에서 시간에 따라 변화할 수 있는 역동적인 특성을 가진다. 체계적 문헌고찰 및 메타분석에서도 노쇠 상태가 악화되는 경우뿐만 아니라 이전 단계로 개선되는 이행이 보고되어 왔다[5]. KFACS의 장기 추적자료를 활용한 분석에서도 노쇠 상태의 유지‧악화‧개선이 모두 관찰되었으며[9], 최근에는 내재역량(intrinsic capacity)의 변화가 노쇠 상태의 지속이나 악화와 관련되는 것으로 나타났다. 특히 이동능력, 활력 및 심리 영역의 저하는 노쇠 상태 악화와 관련되어 다양한 기능 영역을 함께 고려할 필요성이 제시되었다[10].

2024년까지 축적된 8년 추적자료의 내부 분석에서도 장기적인 상태 변화가 확인되었다. 2016년 기반조사와 2024년 추적조사를 비교한 결과 새로운 노쇠 발생률은 11.8%였으며, 노쇠 유병률은 8.8%에서 14.1%로 증가하였다. 8년간 노쇠 상태 이행에서는 50%가 기존 상태를 유지한 반면 33%는 악화되고 17%는 개선되었으며, 전노쇠에서는 노쇠 상태보다 건강한 상태로 회복되는 비율이 상대적으로 높았다[7]. 이러한 결과는 노쇠가 심화되기 전 위험요인을 조기에 파악하고 적절한 예방적 관리가 이루어지는 것이 중요하며, 이는 노쇠의 진행을 예방하거나 지연시킬 가능성이 있음을 보여준다.

#### 2) 근감소증 관련 연구

근감소증은 근육량, 근력 및 신체수행능력의 저하와 관련된 대표적인 노인성 증후군으로 노쇠와 밀접한 관련성을 가진다. KFACS 참여자 2,028명을 분석한 연구에서 Asian Working Group for Sarcopenia 2019 기준의 근감소증 유병률은 17.5%였으며, 노쇠가 동반된 대상자에서 근감소증이나 노쇠만 있는 경우보다 반복 낙상과 골절을 동반한 낙상 등 낙상 관련 건강문제가 더 빈번하게 나타났다[11]. 이는 노쇠와 근감소증이 일부 특성을 공유하면서도 서로 다른 임상적 특성을 갖는다는 점을 보여준다.

KFACS는 국제 근감소증 진단 기준의 적용과 국내 지역사회 노인을 위한 선별‧평가 방법을 검토하는 데에도 활용되었다. European Working Group on Sarcopenia in Older People 기준을 적용한 연구에서는 평가 방법과 임상 알고리즘에 따라 근감소증 유병률에 차이가 나타났으며[12], 종아리 둘레를 활용한 연구에서는 저근육량 선별을 위한 기준으로 남성 35 cm, 여성 33 cm가 제시되었다[13]. 또한 한국어판 SARC-F의 타당도가 검증되었고[14], 5회 의자 일어서기 검사가 근감소증 진단에서 근력과 신체수행능력을 간편하게 평가할 수 있는 보조 지표로 활용될 수 있음을 발표하였다[15].

연구 범위는 근감소성 비만과 신체기능 간의 관련성 및 근감소성 비만의 선별 방법[16,17], 혈액 기반 바이오마커와 근육기능의 관계[18] 등으로 확대되어 왔다. 최근에는 축적된 임상 및 기능자료에 기계학습 기법을 활용한 possible sarcopenia 예측모형을 개발하는 등 근감소증의 조기선별과 예측을 위한 연구도 이루어지고 있다[19]. 이러한 연구들은 KFACS가 근감소증의 역학적 특성뿐 아니라 선별, 위험요인 및 예측 연구를 수행할 수 있는 연구 기반으로 활용되고 있음을 보여준다.

#### 3) 노쇠 관련 주요 건강결과 및 예후 연구

KFACS의 추적자료를 활용하여 노쇠와 이후 발생하는 기능 저하 및 장애 등 주요 건강결과 간의 관계를 분석한 연구가 수행되고 있다. 기반조사와 2년 추적자료에서는 신체적 노쇠가 이후 이동능력과 도구적 일상생활 수행능력(instrumental activities of daily living) 장애 발생과 관련되었으며, 전노쇠 단계에서도 이동능력 장애 위험이 증가하는 것으로 나타났다[20]. 또한 5회 의자 일어서기 검사에서 낮은 수행능력을 보인 대상자는 추적조사에서 보행속도 저하와 기능 제한 등 부정적인 건강결과의 위험이 높아, 신체수행능력 지표의 예후적 활용 가능성이 제시되었다[21]. Timed Up & Go 검사는 신체적 노쇠와 낮은 신체수행능력을 선별하는 간편한 지표로서의 활용 가능성이 확인되었다[22].

KFACS는 인지기능과 의료이용 등 다른 건강 영역의 연구에도 활용되고 있다. 보행속도와 인지기능의 관계를 분석한 연구에서는 느린 보행속도가 낮은 인지기능, 특히 전두엽 관련 기능과 연관되었으며[23], 2년 종단연구에서는 높은 심리적 회복탄력성이 전반적인 인지기능 저하의 정도가 낮은 것과 관련되었다[24]. 또한 KFACS와 국민건강보험 자료를 연계한 연구에서는 2년간 전노쇠에서 노쇠로 악화된 집단에서 건강한 상태를 유지한 집단보다 입원일수와 의료비가 증가하였다[25]. 이러한 연구성과는 KFACS가 신체기능과 장애뿐만 아니라 인지기능 및 의료이용 등 다양한 영역에서 노년기 건강 상태의 변화와 예후를 연구하는 기반으로 활용되고 있음을 보여준다.

### 4. 코호트 연구의 미래

KFACS는 기존 참여자에 대한 장기 추적을 지속하는 동시에 코호트 고령화와 그에 따른 사망, 건강 악화 및 장기요양시설 입소 등으로 인한 추적조사 대상자 감소에 대응하고 변화하는 노년 세대의 특성을 연구하기 위해 2026–2027년에 70–84세 지역사회 거주 노인 약 800명을 신규 모집하고 있다[7]. 신규 참여자에게는 기존 코호트와 유사한 연령 구성과 조사체계를 적용하여, 약 10년의 시차를 두고 노년기에 진입한 두 집단의 노쇠 및 건강 특성을 비교하고 사회‧환경과 생활양식의 변화에 따른 세대효과(generation effect)를 분석할 수 있는 기반을 구축하고자 한다.

기존의 역학‧임상자료와 인체자원 중심의 연구에서 나아가, 음성과 얼굴 영상 등 디지털 바이오마커 자료를 결합하는 방향으로 조사 영역을 확대하고 있다. 최근 연구에서는 음성의 음향학적 특성이 노쇠와 관련될 가능성이 보고되고 있으며, 음성자료에 인공지능을 적용하여 노쇠를 분류하는 연구도 이루어지고 있다[26,27]. 또한 얼굴 영상에서 추출한 표정 특성과 인지장애의 관련성을 분석한 연구와 인공지능 기반 얼굴표정 분석을 활용하여 치매를 조기에 감별하려는 연구가 보고되는 등[28,29] 얼굴 영상의 비침습적 디지털 지표로서의 가치도 주목받고 있다. KFACS에서도 음성과 얼굴 영상에서 추출되는 특성을 기존의 신체‧인지기능 및 임상자료와 연계하여 노쇠 및 기능 저하를 평가‧예측할 수 있는 새로운 디지털 지표로서의 활용 가능성을 탐색하고자 한다.

이러한 연구 확장은 기존 코호트의 장기적인 변화와 신규 세대의 특성을 함께 분석하고, 노쇠의 발생과 진행에 영향을 미치는 요인과 예측 지표를 규명하는 데 기여할 수 있다. 또한 임상‧역학자료, 인체자원 및 디지털 자료를 연계함으로써 노쇠 고위험군의 조기 발견과 예방‧관리 전략 개발을 위한 근거를 축적하고, 이를 통해 세대 간 노쇠 및 건강 특성의 변화를 규명하며, 기존의 임상‧역학자료와 인체자원에 디지털 자료를 연계한 새로운 노화 연구로 연구 영역을 확대할 수 있을 것으로 기대된다.

## 결 론

KFACS는 장기간의 반복 추적을 통해 국내 지역사회 노인의 노쇠 발생과 변화, 관련 위험요인 및 건강결과를 규명할 수 있는 연구 기반을 구축해 왔다. 지속적인 추적조사를 통해 축적된 역학‧임상자료와 인체자원은 노쇠뿐만 아니라 근감소증, 신체‧인지기능 및 다양한 노년기 건강문제를 연구하는 데 폭넓게 활용되고 있다. 향후 기존 참여자의 장기 추적과 신규 대상자 모집을 지속하고 새롭게 구축되는 연구자원을 활용함으로써, 변화하는 우리나라 노인의 건강과 노쇠 양상을 장기적으로 관찰하고 이해할 수 있는 연구 기반으로서의 역할을 이어갈 것으로 기대된다.

## Figures and Tables

**그림 1:** 한국 노인노쇠코호트 연구 참여 10개 센터

**그림 2:** 한국 노인노쇠코호트 추적조사 방법

**그림 3:** 한국 노인노쇠코호트 추적조사 역학 자료와 인체자원 수집 및 관리 ID=identification; OMR=optical mark recognition; DB=database.

**표 1:** 한국 노인노쇠코호트 연구 추적조사 현황 및 추적률

추적조사	연도	조사 대상자(n)	조사 완료자(n)	추적률(%)	사망자(n)
BL	2016	1,558a)	-	-	-
	2017	1,453b)	-	-	-
Wave 2	2018	1,558	1,441	92.5	18
	2019	1,453	1,377	94.8	20
Wave 3	2020	1,540	1,347	87.5	45
	2021	1,433	1,112	77.6	41
Wave 4	2022	1,495	1,135	75.9	105
	2023	1,392	1,106	79.5	63
Wave 5	2024	1,390	994	71.5	115
	2025	1,327c)	991	74.7	93

BL=baseline. ^a)^2016년 기반조사에 등록된 대상자 1,559명 중 1명이 연구 참여 동의 철회하여 조사 대상자 1,558명. ^b)^2017년 기반조사에 등록된 대상자 1,455명 중 2명이 연구 참여 동의 철회하여 조사 대상자 1,453명. ^c)^2025년에 2017년 기반조사 대상자 중 2명이 추가로 연구 참여 동의 철회함. 이전 사망자를 제외한 최종 조사 대상자 1,327명.

**표 2:** 한국 노인노쇠코호트 연구 설문조사 항목

조사 구분	조사 항목
1. 기본사항	대상자 정보, 조사방법(센터방문, 가구방문, 전화조사), 건강보험공단 청구·지급 자료와의 연계를 위한 조사 항목, 주관적 연령
2. 노쇠	CHS 노쇠지표, KLoSHA Frailty Inde (K-IADL, K-ADL), FRAIL, 한국형 노쇠측정도구(KFI), 허약측정도구, SOF Frailty Index, 신체기능제한(PF 척도-mobility), SARC-F, KFS
3. 건강상태	이환, 선행/병용약물, 장기요양보험제도 및 의료이용에 관한 사항, 지역사회 보건 서비스 이용에 관한 사항, 건강정보 이해력, 삶의 질(EQ-5D/EQ-VAS/SF-12), 정신건강(SGDS-K/BRS/LOT-R/UCLA Loneliness Scale/PSS/PSQI/The [Secure] Flourish Index), 낙상, 생활기능
4. 건강행태	흡연, 음주, 신체활동(IPAQ), 영양(MNA/SNAQ/식욕/식생활), 구강건강, 여성건강
5. 사회기능	사회적 관계망(social network), 사회자본(social capital), 사회적 지지(social support), 외출빈도
6. 인지기능(cognitive function)	일반기능(MMSE-KC), 기억력(단어 목록 기억 검사[word list memory], 단어 목록 회상 검사[word list recall], 단어 목록 재인 검사[word list recognition]), 전두엽/실행기능(TMT-A), 주의력(숫자 외우기 검사[digit span]), 전두엽/실행기능(전두엽 기능 평가 [FAB])
6-1. 인지기능(※대리응답인 경우만 시행)	K-AD8 (보호자용)
7. 인구·사회·경제학적 특성	학력 및 총 교육연수, 직업, 결혼 상태 및 독신기간, 가구조사, 국민기초생활보장 수급자 여부, 핸드폰 소유 여부, 주관적 경제수준, 디지털 기기 이해력(MDPQ-16)

CHS=Cardiovascular Health Study; KLoSHA=Korean Longitudinal Study on Health and Aging; K-IADL=Korean Instrumental Activities of Daily Living; K-ADL=Korean Activities of Daily Living; FRAIL=Fatigue, Resistance, Ambulation, Illness, Loss of Weight; KFI=Korean Frailty Index; SOF=Study of Osteoporotic Fractures; PF=Physical Function; SARC-F=Strength, Assistance in walking, Rise from a chair, Climb stairs, and Falls; KFS=Korean Frailty Scale; EQ-5D=EuroQol 5-Dimension; EQ-VAS=EuroQol Visual Analogue Scale; SF-12=12-Item Short Form Health Survey; SGDS-K=Korean version of the Short Form of Geriatric Depression Scale; BRS=Brief Resilience Scale; LOT-R=Life Orientation Test-Revised; UCLA Loneliness Scale=University of California, Los Angeles Loneliness Scale; PSS=Perceived Stress Scale; PSQI=Pittsburgh Sleep Quality Index; IPAQ=International Physical Activity Questionnaire; MNA=Mini Nutritional Assessment; SNAQ=Simplified Nutritional Appetite Questionnaire; MMSE-KC=Mini-Mental Status Examination, Korean version of CERAD Assessment Packet; TMT-A=Trail Making Test A; FAB=Frontal Assessment Battery; K-AD8=Korean version of the Ascertain Dementia 8-item Informant Questionnaire; MDPQ-16=Mobile Device Proficiency Questionnaire.

**표 3:** 한국 노인노쇠코호트 연구 검진조사 항목

조사 구분	조사 항목
신체검진	
1. 체형	신장/체중/허리둘레/상완위 둘레/종아리 둘레/핑거링 테스트
2. 활력징후	혈압(수축기/이완기)/맥박
3. 시력, 청력	시력 - 원거리 시력/근거리 시력/근거리 입체시 청력 - 500/1,000/2,000/4,000 Hz
4. 채혈	공복혈당(FBS), 인슐린, 당화혈색소(HbA1c), 총단백질, 알부민, BUN, creatinine, GOT (AST), GPT (ALT), GGT (γ-GTP), 총콜레스테롤, HDL 콜레스테롤, LDL 콜레스테롤, 중성지방, uric acid, WBC, RBC, Hb, Hct, platelet, MCV, MCH, MCHC, (2024년부터 추가 분석: hs-CRP, cystatin C)
5. 채뇨	pH, nitrite, S.G, 단백질, 포도당, 케톤, 빌리루빈, 잠혈(occult blood), 유로빌리노겐, 백혈구 에스테라제
6. ECG	
7. 흉부 X-선 검사	
8. 구강노쇠	구강노쇠 위험도 선별 문진표(12문항)
9. 신체조성	DXA/BIA
10. 골다공증 검사	DXA 대퇴골과 요추(2024년부터 추가 검진)
신체기능검사	
1. 악력	
2. 단축형 신체기능평가(SPPB)	밸런스 검사(일반자세/반 일렬자세/일렬자세) 보행 속도 - 4 m 걸어가기(가속구간 & 감속구간): 센서기 측정/초시계 측정 - 4 m 걸어가기(가속구간 & 감속구간 없음): 센서기 측정/초시계 측정 - 2.4 m 걸어가기(가속구간 & 감속구간 없음): 초시계 측정(가구방문조사) 의자 앉았다 일어서기 - 초시계 측정
3. Timed Up & Go test (3 m 걸어 돌아오기)	
4. 각근력	무릎관절 신전력 1, 2차 측정/무릎관절 굴곡력 1, 2차 측정
5. 영상보행 측정	
6. 음성인식 측정	구강노쇠/문장구조 발화/자유 발화(그림 묘사)
7. 영상촬영 측정	정지 영상/발화 영상

FBS=fasting blood sugar; HbA1c=hemoglobin A1c; BUN=blood urea nitrogen; GOT=glutamic oxaloacetic transaminase; AST=aspartate aminotransferase; GPT=glutamic pyruvic transaminase; ALT=alanine aminotransferase; GGT=gamma-glutamyl transferase; γ-GTP=gamma-glutamyl transpeptidase; HDL=high-density lipoprotein; LDL=low-density lipoprotein; WBC=white blood cell; RBC=red blood cell; Hb=hemoglobin; Hct=hematocrit; MCV=mean corpuscular volume; MCH=mean corpuscular hemoglobin; MCHC=mean corpuscular hemoglobin concentration; hs-CRP=high-sensitivity C-reactive protein; S.G=specific gravity; ECG=electrocardiography; DXA=dual-energy X-ray absorptiometry; BIA=bioelectrical impedance analysis; SPPB=Short Physical Performance Battery.

**표 4:** 추적조사 방법에 따른 조사 항목 요약

조사방법	조사유형	본인응답(self-report)	대리응답(proxy)
센터방문	설문조사	설문 모두 시행	설문 일부 시행(대상자와 동거 여부, 대상자와 관계, 대상자 현재상태, 일상생활활동 측정도구[K-IADL], FRAIL, 낙상, 건강행태[흡연, 음주, 신체활동, 영양], 사회기능[사회자본], 인지기능[K-AD8], 인구‧사회‧경제학적 특성[결혼상태, 가구형태, 가구수입])
	검진조사	검진 모두 시행	-
가구방문	설문조사	설문 모두 시행	센터방문 대리응답자 항목과 동일함
	검진조사	검진 일부 시행(체형[신장, 체중, 신체 둘레], 활력징후[혈압/맥박], 시력 및 청력, 채혈, 신체기능[악력, SPPB])	-
전화조사	설문조사	설문 일부 시행(일상생활활동 측정도구[K-IADL, K-ADL], FRAIL, 낙상, 신장, 현재 체중, 1년 전의 체중)	설문 일부 시행(대상자와 동거 여부, 대상자와 관계, 대상자 현재 상태, 일상생활활동 측정도구(K-IADL), FRAIL, 낙상, 인지기능[K-AD8])
	검진조사	-	-

K-IADL=Korean Instrumental Activities of Daily Living; FRAIL=Fatigue, Resistance, Ambulation, Illness, Loss of Weight; K-AD8=Korean version of the Ascertain Dementia 8-item Informant Questionnaire; SPPB=Short Physical Performance Battery; K-ADL=Korean Activities of Daily Living.
